# Successful MRI-Guided Focused Ultrasound Uterine Fibroid Treatment Despite an Ostomy and Significant Abdominal Wall Scarring

**DOI:** 10.5402/2011/962621

**Published:** 2010-10-17

**Authors:** Ronit Machtinger, Clare M. C. Tempany, Angela Kanan Roddy, Fiona M. Fennessy

**Affiliations:** ^1^Department of Obstetrics and Gynecology, Brigham and Women's Hospital, Harvard Medical School, 45 Francis Street, Boston, MA 02115, USA; ^2^Department of Radiology, Brigham and Women's Hospital, Harvard Medical School, 45 Francis Street, Boston, MA 02115, USA

## Abstract

We present a case of successful magnetic resonance imaging-guided focused ultrasound surgery (MRgFUS) of a uterine fibroid in a patient with extensive anterior abdominal wall surgical scars from two longitudinal laparotomies, a total colectomy and ileostomy. This case demonstrates that MRgFUS can be safely used in patients with an ostomy and significant abdominal wall scarring, but careful pretreatment planning and positioning during treatment is needed.

## 1. Case Report

MRI-guided focused ultrasound (MRgFUS) is a noninvasive method of thermal ablation, which, through MRI guidance, allows for 3D treatment planning and feedback of temperature deposition in the area to be treated. MRgFUS was FDA approved for fibroid treatments in 2004 [1, 2]. Obstruction in the near-field of the focused ultrasound beam, such as from an ostomy bag, or indeed extensive abdominal wall scar tissue (which has different acoustic properties), could lead to increased absorption of acoustic energy and skin burns [3]. We discuss MRgFUS of a symptomatic uterine fibroid in a patient with longitudinal abdominal scarring and ileostomy and discuss how we circumnavigated these potential obstructions.

A nulliparous 48-year-old premenopausal woman with a symptomatic uterine fibroid presented for MRgFUS. She had menometrorrhagia, bulk symptoms, urinary frequency, and fatigue. Her fibroid symptom severity score and health-related quality of life questionnaire (transformed UFS-SSS QOL) [4–7] was 93.8 (on a scale of 0–100), indicating very symptomatic disease. She had declined hysterectomy, having already had two laparotomies in the past for total colectomy and ileoanal pouch formation for inflammatory bowel disease, and subsequent lysis of adhesions and ileostomy. She was fearful of possible complications related to adhesions and pelvic floor compromise should she undergo additional surgery. In addition, as a self-employed woman, she was most interested in a minimally invasive intervention requiring the least amount of recovery time. 

On physical examination, there was a right lower quadrant ileostomy and a well-healed vertical scar extending from above the umbilicus to the mons pubis. A firm, nontender pelvis mass in the left lower quadrant was appreciated up to the level of the umbilicus. Diagnostic MRI demonstrated the uterus measuring 9.7 × 10.0 × 9.5 cm with a single anterior intramural fibroid measuring 8.8 × 7.6 × 6.4 cm. The fibroid was homogenous and isointense to muscle on the T1-weighted images and of low signal intensity on the T2-weighted images and demonstrated mostly homogeneous enhancement post intravenous gadolinium injection.

**Figure 1:**
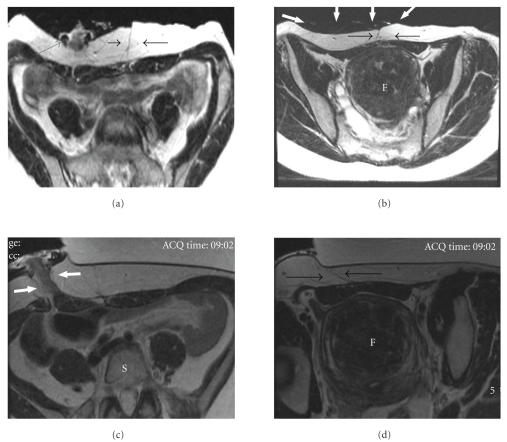
Pretreatment screening axial T2 weighted screening MR images (a, b) and axial T2 weighted images on the day of treatment (c, d). On screening images, the ostomy is evident in the right lower quadrant (grey arrows), and longitudinal abdominal scarring is seen in the subcutaneous tissue in the midline (black arrows) which overlies the uterine fibroid (F). The ostomy bag can also be seen to overlie the anterior abdominal wall (white thick arrows). On the day of treatment, the ostomy bag was placed off to the side and was no longer visible on the images. The skin of the anterior abdominal wall was pulled off to the right side such that the ostomy and scar are now out of the treatment window and the fibroid (F) is accessible for treatment.

After review of the screening MR images (Figures 1(a) and 1(b)) and consultation with the hospital ostomy service, a plan was made to attach the ostomy bag off-center, cut back the wafer/base plate, and rotate the bag off to the side such that it lay away from the center of the abdomen. The patient was asked to fast from midnight the night before and to maintain a liquid diet in the 24 hours prior to treatment to decrease ostomy output.

On the day of the procedure, the patient was positioned prone and slightly off-center on the MRI table, such that the left lower anterior wall was positioned over the gel pad and the ostomy off to the right side and out of the treatment path. The skin of the anterior abdominal wall was pulled towards the ostomy, such that the midline longitudinal scars were also out of the treatment field, increasing the size of the acoustic or treatment window and subsequently allowing treatment of a greater fibroid volume (Figures 1(c) and 1(d)). 

**Figure 2:**
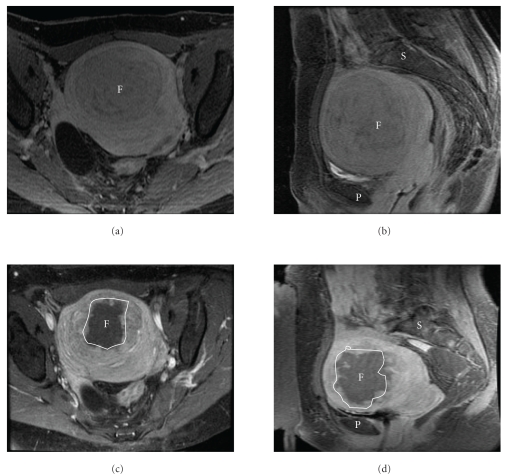
T1 spoiled gradient recalled (SPGR) echo sequences in the axial (a, c) and sagittal (b, d) planes, post administration of intravenous gadolinium gadopentate. Imaging acquired pretreatment (a, b) demonstrates homogenous enhancement of the fibroid (F), and post treatment (c, d) there is a large area of nonenhancement within the fibroid (outlined in white), consistent with a treatment effect. There is no evidence for enhancement in the scar tissues of the anterior abdominal wall. (P): Pubic bone; (S): spine.

During treatment, as always, careful attention was paid to the thermal maps to ensure no heat builds up outside of the fibroid. Following delivery of multiple high power sonications to the treatment area, intravenous gadolinium was administrated and showed a 4.1 × 5 × 4.7 cm area of nonenhancement, consistent with necrosis, within the fibroid. No abnormal areas of enhancement within the subcutaneous tissue or the regions of the scar were identified (Figures 2(a)–2(d)). 

Three months post treatment, the patient reported marked symptom improvement with a decrease in bulk symptoms and increase in energy. Her transformed UFS-SSS QOL decreased from 93.8 to 50. No skin changes, abnormality, or any changes in ostomy function were reported.

## 2. Discussion

To our knowledge, this case report is the first to describe MRgFUS in a patient with an ostomy and a history of multiple abdominal surgeries. This case posed a number of potential challenges: the presence of an ostomy, longitudinal abdominal scarring and multiple abdominal surgeries, which pose a threat of underlying adhesions. MRgFUS treatment of patients with horizontal scars [8], usually secondary to cesarean sections or myomectomies [8], can be managed by angling the ultrasound beam superior or inferior to the horizontal Pfannenstiel scar. However, longitudinal scars are more problematic as they are usually midline and the beam has to penetrate from either side of a usually extensive scar. 

This case demonstrates how thorough individualized, patient-specific planning is paramount to a successful treatment. The presence of an overlying ostomy bag and stoma, coupled with longitudinal scars, are not a contraindication to successful MRgFUS fibroid treatment, which is a treatment option to be considered by patients with such medical problems.
